# Growth phase-dependent expression profiles of three vital H-NS family proteins encoded on the chromosome of *Pseudomonas putida* KT2440 and on the pCAR1 plasmid

**DOI:** 10.1186/s12866-017-1091-6

**Published:** 2017-08-29

**Authors:** Zongping Sun, Delyana Vasileva, Chiho Suzuki-Minakuchi, Kazunori Okada, Feng Luo, Yasuo Igarashi, Hideaki Nojiri

**Affiliations:** 1 Research Center of Bioenergy & Bioremediation, College of Resources and Environment, Southwest University, No.2 Tiansheng Road, BeiBei District, Chongqing, 400715 China; 2 Biotechnology Research Center, The University of Tokyo, 1-1-1 Yayoi, Bunkyo-ku, Tokyo, 113-8657 Japan

**Keywords:** H-NS family proteins, Nucleoid-associated proteins, Plasmid, *Pseudomonas*

## Abstract

**Background:**

H-NS family proteins are nucleoid-associated proteins that form oligomers on DNA and function as global regulators. They are found in both bacterial chromosomes and plasmids, and were suggested to be candidate effectors of the interaction between them. TurA and TurB are the predominantly expressed H-NS family proteins encoded on the chromosome of *Pseudomonas putida* KT2440, while Pmr is encoded on the carbazole-degradative incompatibility group P-7 plasmid pCAR1. Previous transcriptome analyses suggested that they function cooperatively, but play different roles in the global transcriptional network. In addition to differences in protein interaction and DNA-binding functions, cell expression levels are important in clarifying the detailed underlying mechanisms. Here, we determined the precise protein amounts of TurA, TurB, and Pmr in KT2440 in the presence and absence of pCAR1.

**Results:**

The intracellular amounts of TurA and TurB in KT2440 and KT2440(pCAR1) were determined by quantitative western blot analysis using specific antibodies. The amount of TurA decreased from the log phase (~80,000 monomers per cell) to the stationary phase (~20,000 monomers per cell), while TurB was only detectable upon entry into the stationary phase (maximum 6000 monomers per cell). Protein amounts were not affected by pCAR1 carriage. KT2440(pCAR1pmrHis), where histidine-tagged Pmr is expressed under its original promotor, was used to determine the intracellular amount of Pmr, which was constant (~30,000 monomers per cell) during cell growth. Quantitative reverse transcription PCR demonstrated that the transcriptional levels of *turA* and *turB* were consistent with protein expression, though the transcriptional and translational profiles of Pmr differed.

**Conclusion:**

The amount of TurB increases as TurA decreases, and the amount of Pmr does not affect the amounts of TurA and TurB. This is consistent with our previous observation that TurA and TurB play complementary roles, whereas Pmr works relatively independently. This study provides insight into the molecular mechanisms underlying reconstitution of the transcriptional network in KT2440 by pCAR1 carriage.

**Electronic supplementary material:**

The online version of this article (doi:10.1186/s12866-017-1091-6) contains supplementary material, which is available to authorized users.

## Background

Nucleoid-associated proteins (NAPs) play important roles in chromatin compaction and global regulation in bacterial cells [1–3]. Among the best characterized NAPs are the histone-like protein H1 (H-NS) family proteins. They have two structurally independent domains connected by a flexible linker, an N-terminal domain involved in dimerization/oligomerization, and a C-terminal domain in charge of DNA-binding [4]. MvaT homologs of *Pseudomonas* were experimentally shown to complement *Escherichia coli* H-NS-deficient phenotypes, and are now recognized as members of the H-NS family despite low sequence similarity [5]. *Pseudomonas putida* KT2440 harbors five genes encoding H-NS family proteins, namely PP_1366 (TurA), PP_3765 (TurB), PP_0017 (TurC), PP_3693 (TurD), and PP_2947 (TurE) [6]. Our previous report revealed that several bacteria carry the same types of H-NS family proteins on both their plasmids and chromosome [7]. An MvaT homolog, Pmr, is encoded on the carbazole-degradative incompatibility (Inc) P-7 group plasmid pCAR1, whose hosts are mainly *Pseudomonas* [8–12].

*turA* and *turB* are predominantly transcribed in log and stationary phases, respectively, in KT2440 and KT2440(pCAR1) cells, whereas *pmr* is actively transcribed in KT2440(pCAR1) cells [13]. TurA, TurB, and Pmr can form homo- and hetero-oligomers in vitro based on the N-terminal domain, while the coupling ratios among them differ [14, 15]. In addition, though the TurA-, TurB-, and Pmr-binding regions detected in vivo were almost identical, the regulons of the three proteins vary significantly [16]. While pCAR1 carriage altered the global transcriptional network in KT2440 cells [17–19], our previous results suggested that the three H-NS family proteins function cooperatively, but their respective roles are not equivalent [16]. To understand the molecular mechanisms underlying reconstitution of the transcriptional network in KT2440 by pCAR1 carriage, it is important to determine the detailed roles of these proteins.

To this end, in addition to the DNA-binding and dimerization/oligomerization functions, it is important to determine the intracellular amounts of the three H-NS family proteins and how they cooperatively compact the genome and regulate gene expression. The transcriptional levels of *turA*, *turB*, and *pmr* were previously determined [13], but the transcriptional and translational levels sometimes differ among H-NS family proteins [20]. In addition, NAPs show various expression patterns throughout the growth phases [21]. Thus, in the present study, we quantified the intracellular amounts of TurA, TurB, and Pmr in KT2440 and/or KT2440(pCAR1) cells during the growth phases by western blot analysis using specific antibodies. We provide basic knowledge about the cooperative regulatory network of the H-NS family proteins in KT2440 and KT2440(pCAR1) cells.

## Methods

### Bacterial strains, plasmids, and media

Bacterial strains and plasmids used in this study are listed in Table 1. *E. coli* BL21(DE3), used for the overexpression of histidine (His)-tagged TurA, TurB, and Pmr, was cultured in lysogeny broth (LB) [22] at 25 or 30 °C. *E. coli* DH5α, used for plasmid construction, was cultured in LB at 37 °C. *Pseudomonas* strains were cultured at 30 °C in LB filtered by Stericups, 0.22-μm pore size filters (Merck Millipore, Darmstadt, Germany). The medium was supplemented with 50 μg/mL kanamycin (Km) or 30 μg/mL chloramphenicol (Cm) where necessary. Solid medium was prepared by the addition of 1.6% (*w*/*v*) agar powder (Nacalai Tesque, Kyoto, Japan).

**Table 1:** Bacterial strains and plasmids used in this study

Strain or plasmid	Relevant characteristics	Source or reference
Bacterial strains		
*Escherichia coli*		
BL21(DE3)	F¯ *ompT hsdS*(r_B_¯ m_B_¯) *gal dcm* λ (DE3)	Novagen
DH5α	F¯ ϕ80d*lacZ*ΔM15 Δ(*lacZYA*-*argF*)*U169 endA1 recA1 hsdR17*(r_K_¯m_K_^+^) *deoR thi-1 supE44 gyrA96 relA1 λ* ^*-*^ *phoA*	Toyobo
*Pseudomonas putida*		
KT2440	Naturally Cm^r^	[34]
KT2440(pCAR1)	KT2440 harboring pCAR1	[17]
KT2440(pCAR1pmrHis)	KT2440(pCAR1) carrying gene encoding His-tagged Pmr under original promotor	[13]
Plasmids		
pET-C-His-turA	pET-26b(+) with NdeI-XhoI fragment containing *turA*	[14]
pET-C-His-turB	pET-26b(+) with NdeI-SalI fragment containing *turB* ligated into the NdeI-XhoI site	[14]
pET-C-His-pmr	pET-26b(+) with NdeI-XhoI fragment containing *pmr*	[13]
pT7Blue T-vector	Ap^r^, *lacZ*α, T7 promoter, f1 origin, pUC/M13 priming sites	Novagen
pTuniv16S	pT7Blue T-vector with PCR fragment amplified from total DNA of *Pseudomonas resinovorans* CA10 with the primer set, univ16S-F and univ16S-R.	[19]
pTturA	pT7Blue T-vector with PCR fragment amplified from total DNA of KT2440 with the primer set, PP_1366-F and PP_1366-R.	This study
pTturB	pT7Blue T-vector with PCR fragment amplified from total DNA of KT2440 with the primer set, PP_3765-F and PP_3765-R.	This study
pTpmr2	pT7Blue T-vector with PCR fragment amplified from pET-C-His-pmr with the primer set, pmr-F-2 and pmr-R-2.	This study

### Determination of total cell number

To cultivate *P. putida* strains, a single colony from an overnight-incubated LB agar plate was inoculated into 5 mL of fresh LB for pre-cultivation. When pCAR1-harboring strains were used, the carbazole-degrading ability of the cells was confirmed with 0.1% carbazole-containing minimal agar [23] before pre-cultivation. After overnight pre-cultivation, the second pre-cultivation was performed in 100 mL of LB. Optical density at 600 nm (OD_600_) was adjusted to 0.03 and cells were incubated for 4 h. Then the portion of the resultant culture was transferred into 400 mL of fresh LB, where the OD_600_ was re-adjusted to 0.03, and cultivation was started. The OD_600_ was monitored and cells were harvested every 2 h after inoculation. At the same time, aliquots (0.5 mL) of the culture were harvested to determine the total number of cells. The aliquots were mixed with an equal volume of 0.3% (*w*/*v*) crystal violet and a volume of PBS buffer (2.56 g/L Na_2_HPO_4_.7H_2_O, 8 g/L NaCl, 0.2 g/L KCl, 0.2 g/L KH_2_PO_4_, pH 7.4) was added for appropriate dilution. Ten microliters of the mixture were applied to a C-Chip DHC-N01 hemocytometer (NanoEnTek, Seoul, Korea) to count the number of cells using an optical microscope (BX53; Olympus, Tokyo, Japan).

### Expression and purification of TurA, TurB, and Pmr

TurA, TurB, and Pmr were overexpressed as C-terminal His-tagged forms using pET-C-His-turA, pET-C-His-turB, and pET-C-His-pmr, respectively, and purified to homogeneity as described previously [14]. These proteins were used as standards in western blot analysis.

### Preparation of antibodies

Polyclonal antibodies against TurA and TurB were prepared by Sigma-Aldrich (St. Louis, MO, USA). The antibodies were produced in rabbits by injecting synthesized peptides corresponding to residues 61–77 of TurA and 12–29 of TurB. The resultant serum was used in western blot analysis. Anti-His antibody (Medical and Biological Laboratories, Nagoya, Japan) was used for detection of C-terminal-His-tagged Pmr in KT2440(pCAR1pmrHis).

### Preparation of whole cell protein for quantification of TurA, TurB, and His-tagged Pmr

Preparation of whole cell protein was performed according to the methods of Azam et al. and Ohniwa et al. [21, 24] with modifications. KT2440, KT2440(pCAR1), or KT2440(pCAR1pmrHis) cells (10^9^–10^10^) were harvested by centrifugation at various time intervals and washed with PBS buffer. Cell pellets were suspended in 250 μL of Buffer A containing 40 mM Tris-HCl (pH 8.1) supplemented with 25% sucrose, followed by the addition of 50 μL of Buffer B (40 mM Tris-HCl [pH 8.1], 10 mM EDTA [pH 8.1], and 3 mg/mL lysozyme) and incubated for 20 min on ice. Lysis was then performed by addition of 100 μL of Buffer C (10 mM Tris-HCl [pH 8.2], 10 mM EDTA [pH 8.0], 1% Brij 58, and 0.4% sodium deoxycholate) in the presence of 0.75% detergent NP-40 and 0.5 mM phenylmethylsulfonyl fluoride for 20 min on ice. The mixtures were supplemented with 50 μL of 2 M KCl, 2.5 μL of 2 M MgCl_2_, 10 μg of RNase, and 12.5 μg of DNaseI and incubated for 10 min at 37 °C. Finally, sonication was performed for 10 min (10 s on/30 s off; output power, high-level) using a Bioruptor II (Cosmo Bio, Tokyo, Japan). Protein concentration was determined using Protein Assay Dye Reagent Concentrate (Bio-Rad Laboratories, Hercules, CA, USA).

### Western blot analysis

To confirm the specificity of anti-TurA and anti-TurB antibodies, purified His-tagged TurA, TurB, and Pmr were electrophoresed on a 15% tricine-SDS-PAGE gel and then transferred onto a Sequi-Blot polyvinylidene difluoride (PVDF) membrane (Bio-Rad Laboratories) at 4 °C, overnight. The blot was blocked at room temperature with 5% enhanced chemiluminescence (ECL) blocking agent (GE Healthcare, Little Chalfont, UK) in TBST buffer (10 mM Tris-HCl [pH 8.0], 0.146 M NaCl, 0.05% Tween-20) for 1 h, washed with TBST (three times for 5 min each), and then incubated with 5000-fold diluted anti-TurA or 1000-fold diluted anti-TurB antibodies in TBST supplemented with 5% ECL blocking agent for 1 h at room temperature. After an additional washing step with TBST (three times for 15 min each), the blot was incubated with a horseradish peroxidase-conjugated goat anti-rabbit secondary antibody (Sigma-Aldrich; 10,000-fold diluted in TBST containing 5% ECL blocking agent) for 1 h at room temperature. After an additional washing step with TBST (three times for 15 min each), the blot was probed with 6 mL of Immobilon Western Chemiluminescent HRP Substrate (Merck Millipore) for 5 min and then detected using LAS1000 (Fujifilm, Tokyo, Japan) image analyzer.

To quantify TurA and TurB, whole cell lysates containing 80 μg of protein from KT2440 and KT2440(pCAR1) were separated by 13% glycine-SDS-PAGE. To quantify His-tagged Pmr, whole cell lysate containing 40 μg of protein from KT2440(pCAR1pmrHis) was separated by 13% glycine-SDS-PAGE. Different amounts of purified His-tagged TurA, TurB, or Pmr, for which a linear relationship between protein concentration and signal intensity had been confirmed, were used as standards in each assay. PVDF membrane transfer and development of the blots were performed essentially as described above, except that the dilution ratio of anti-TurA, anti-TurB, and anti-His antibodies were 3000-fold, 2000-fold, and 5000-fold, respectively. The images were obtained using LAS1000 or LAS500 (GE Healthcare) image analyzers. The intensity of the protein bands was determined using ImageJ software (http://rsbweb.nih.gov/ij/). RNA polymerase α subunit was employed as a control for sample loading. Anti-RNA polymerase α subunit antibody (NeoClone, Madison, WI, USA; 2000-fold dilution in TBST with 5% ECL blocking agent) and ECL peroxidase-labeled anti-mouse antibody (GE Healthcare; 10,000-fold dilution in TBST with 5% ECL blocking agent) were used as primary and secondary antibodies, respectively, with 5 μg of whole cell protein samples.

### RNA extraction and cDNA synthesis

RNA extraction was performed according to a previous report [19]. First-strand cDNA synthesis was conducted with 1.25 μg of purified total RNA, 250 ng of random primers (Invitrogen, Carlsbad, CA, USA), 1 × First Strand Buffer (Invitrogen), 40 U of RNase OUT (Invitrogen), 5 mM dithiothreitol (Invitrogen), and 200 U of SuperScript III Reverse Transcriptase (Invitrogen). After the RNA and random primers had been denatured at 65 °C for 5 min and annealed at 4 °C for 2 min, the remaining reagents were added, and the mixture was incubated at 25 °C for 5 min, 50 °C for 60 min, and 70 °C for 15 min. To degrade the RNA template, 6.67 μL of 1 N NaOH were added and the reaction mixture was incubated at 65 °C for 30 min, then the mixture was neutralized with 6.67 μL of 1 N HCl.

### Quantitative reverse transcription (qRT)-PCR

qRT-PCR was performed using Power SYBR green PCR master mix (Applied Biosystems, Foster City, CA, USA) and the ABI 7300 real-Time PCR System (Applied Biosystems) according to a previous method [19] with some modifications. The primers used for qRT-PCR are shown in Table 2. The reaction conditions were as follows: 95 °C for 10 min for enzyme activation and 40 cycles of 95 °C for 5 s, 60 °C for 10 s, and 72 °C for 35 s. A melting-curve analysis was performed to verify the amplification specificity. For a standard curve, a series of 10-fold dilutions of the target PCR product ligated into the pT7Blue T-vector (Novagen, Madison, WI, USA) were used. 16S rRNA was used as an internal standard. All of the reactions were carried out at least in triplicate, and the data were normalized using the average of the internal standard.

**Table 2:** Primers used for qRT-PCR

Name	Sequences (5′→3′)	References
univ16S-F	ACACGGTCCAGACTCCTACG	17
univ16S-R	TACTGCCCTTCCTCCCAACT	17
PP_1366-F	AACTGGAGTTCGAAGGCAAA	13
PP_1366-R	GAGGTGCCTTGCTCAGTTTC	13
PP_3765-F	ATATCATCGCCATCCTCGAC	13
PP_3765-R	TGCGGGTTCTGATAGACCTT	13
pmr-F-2	TCGCGATTCTTGATCCGGAC	This study
pmr-R-2	CCTTGGTCTCAACGAGCTCA	This study

## Results and discussion

### Design of anti-TurA and anti-TurB antibodies and confirmation of their specificity

**Fig. 1:**
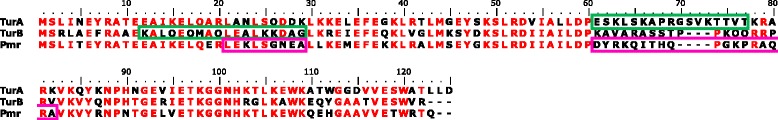
Amino acid sequence alignment of TurA, TurB, and Pmr. The alignment was performed using the ClustalW program (version 2.1, http://clustalw.ddbj.nig.ac.jp/). Identical amino acids between at least two proteins are shown in red. Residues 61–77 of TurA and residues 12–29 of TurB (highlighted in green boxes) were used as antigens to produce the anti-TurA and anti-TurB antibodies used in this study. Residues 21–29 and 61–78 of Pmr (highlighted in the pink boxes) were also used as antigens to produce anti-Pmr antibody, but failed to detect Pmr in KT2440 (pCAR1) cell lysates (data not shown)

**Fig. 2:**
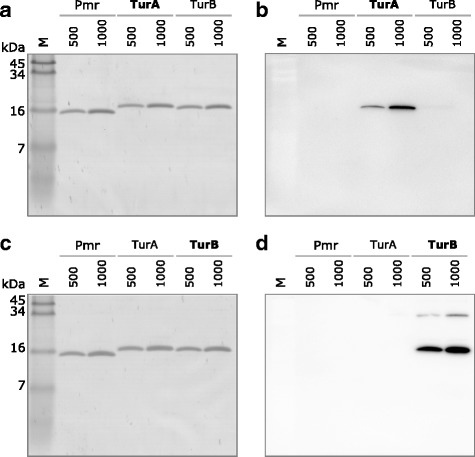
Confirmation of anti-TurA and anti-TurB antibody specificity with purified His-tagged TurA, TurB, and Pmr. Panels **a** and **c** show the results of tricine-SDS-PAGE analyses of 500 or 1000 ng of purified TurA, TurB, and Pmr, where M represents the protein mass marker. Panels **b** and **d** show the results of western blotting using anti-TurA (**b**) and anti-TurB antibodies (**d**). Note that the gels shown in panels **a** and **c** were used for western blotting in panels **b** and **d**, respectively

Considering that the three MvaT homologs have high amino acid sequence identity (50–60%), peptides that show relatively low identity should be used as antigens to avoid cross-reaction. The highlighted sequences of TurA and TurB shown in Fig. 1 (indicated by the green boxes) were used for the preparation of anti-TurA and anti-TurB antibodies. Specificity of these antibodies was confirmed with purified His-tagged TurA, TurB, and Pmr using western blotting (Fig. 2).

We also produced anti-Pmr antibodies using the sequences indicated by the pink boxes in Fig. 1, but failed to detect Pmr in KT2440(pCAR1) cell lysates (data not shown). Thus, we used the KT2440(pCAR1pmrHis) [13] strain, where His-tagged Pmr is expressed under its original promotor, with specific anti-His antibody to quantify the intracellular amount of Pmr. Note that there was little possibility of the C-terminal His-tags affecting the DNA-binding ability, sequence preference, or three-dimensional structure of Pmr. The structure of MvaT, an H-NS family protein of *Pseudomonas aeruginosa* PAO1, determined by NMR clearly suggested that the C-terminus is not involved in DNA-binding [25]. In addition, binding sites of His-tagged Pmr were successfully determined by modified chromatin precipitation assay coupled with microarray technology using the same strain [13, 16]. Therefore, the His-tag introduced at the C-terminus is expected to have a negligible effect on the function of Pmr.

### Intracellular amounts of TurA, TurB, and his-tagged Pmr

**Fig. 3:**
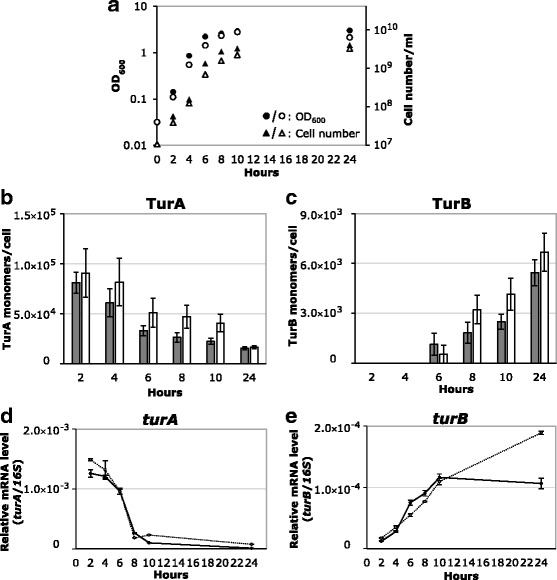
Quantification of TurA and TurB monomers per cell in KT2440 and KT2440(pCAR1) cells. Panel **a** shows the growth curves of KT2440 and KT2440(pCAR1) in LB. The OD_600_ of the culture was measured every 2 h (circles) and cell number per milliliter was counted at the same time (triangles). Filled symbols represent the results of KT2440 and open symbols represent those of KT2440(pCAR1). In panels **b** and **c**, the numbers of TurA (**b**) and TurB (**c**) monomers per cell are shown. Values and error bars correspond to averages and standard deviations of results from at least three independent biological replicates. Gray bars represent the results of KT2440 and white bars represent those of KT2440(pCAR1). Note that the amount of TurB was below the detection limit at 2 h and 4 h in both KT2440 and KT2440(pCAR1). In panels **d** and **e**, corresponding mRNA levels of *turA* (**d**) and *turB* (**e**) genes are shown. Values and error bars correspond to averages and standard deviations of results from at least three independent technical replicates. Filled symbols and lines represent the results of KT2440 and open symbols and dot lines represent those of KT2440(pCAR1)

**Fig. 4:**
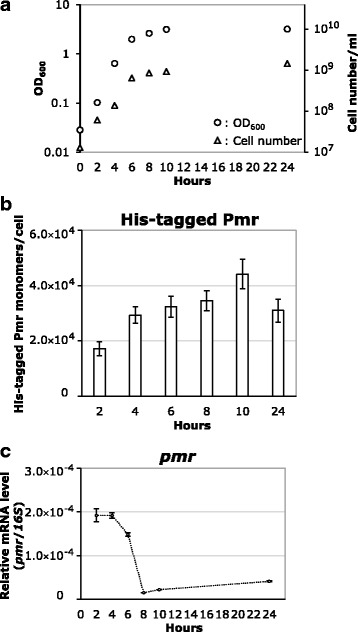
Quantification of His-tagged Pmr monomers per cell in KT2440(pCAR1pmrHis) cells. Panel **a** shows the growth curve of KT2440(pCAR1pmrHis) in LB. The OD_600_ (circles) and cell number per milliliter (triangles) of the culture were measured every 2 h. Panel **b** shows the number of His-tagged Pmr monomers per cell. Values and error bars correspond to averages and standard deviations of results from at least three independent biological replicates. Panel **c** shows the corresponding mRNA levels of the *pmr* gene. Values and error bars correspond to averages and standard deviations of results from at least three independent technical replicates

To quantify the intracellular amount of the three H-NS family proteins using western blotting, whole cell protein extraction was performed with KT2440, KT2440(pCAR1), and KT2440(pCAR1pmrHis) cells cultured in LB. Three independent biological replicates were prepared to quantify total amounts of DNA-bound and free-state TurA, TurB, and His-tagged Pmr. The representative western blot raw data are provided as Additional file 1. A loading control using anti-RNA polymerase α subunit antibody was included to ensure that equal amounts of protein were loaded in each lane. As the growth of the cultures was monitored by OD_600_ measurement and cell counting (Figs. 3a and 4a), the intracellular amounts of the proteins of interests could be calculated per cell. RNA extraction was conducted with cells from the same culture at the same sampling time to investigate the relationship between transcription and translation.

TurA amount declined from ~80,000 monomers per cell at the early log phase to ~20,000 monomers per cell at the late stationary phase in both KT2440 and KT2440(pCAR1) (Fig. 3b). A relatively rapid decrease was observed in the early stationary phase: The number of TurA monomers was reduced almost by half during the first 6 h of cultivation. This tendency was similar to the transcriptional profile of *turA* (Fig. 3d). In contrast, TurB was below the detection limit during the log phase in both KT2440 and KT2440(pCAR1), but was detected upon entry into the stationary phase (Fig. 3c). The intracellular number of TurB monomers increased from ~1000 (6 h) to ~6000 (24 h) per cell. Transcriptional levels of the *turB* gene similarly increased toward the stationary growth phase (Fig. 3e). Note that the transcriptional profiles of *turA* and *turB* in KT2440 and KT2440(pCAR1) were consistent with previous reports [13, 26]. TurA is present mainly in cells during log phase, whereas both TurA and TurB are present in cells during the stationary phase. pCAR1 carriage did not lead to obvious differences in the transcriptional and translational levels of TurA and TurB in KT2440.

Similarly, we determined cell growth and intracellular amounts of His-tagged Pmr in KT2440(pCAR1pmrHis) (Fig. 4). As was shown in Fig. 4b, the His-tagged Pmr amount was relatively constant during growth at around 30,000 monomers per cell, with some fluctuations. These results differ from those in our previous study, in which the band intensity of His-tagged Pmr increased from 2 to 10 h after inoculation [13]. Because the expression levels of His-tagged Pmr were monitored in western blots of soluble proteins in our previous study, we consider the current results to be more accurate. The transcriptional profile of *pmr* detected in this study revealed a rapid decrease in *pmr* transcription at around the transition state to stationary phase (Fig. 4c), a similar pattern to that described by Yun et al. [13]. Notably, while the transcription of *pmr* decreased rapidly 8 h after inoculation, Pmr protein levels were stable in KT2440(pCAR1pmrHis) cells until the late stationary phase. This tendency differs from the chromosomally-encoded TurA protein (Fig. 3). In the case of Sfh, an H-NS family protein encoded on the IncHI1 self-transmissible plasmid pSf-R27 originally found in *Shigella flexneri* serotype 2a strain 2457T, the *sfh* transcription level decreased to nearly 60% in the early stationary phase while the Sfh protein increased almost 2.5-fold as the culture entered the stationary phase [20]. The results in this study suggest the possibility that the protein levels of Pmr and Sfh are subject not only to transcriptional but also post-transcriptional control, such as degradation. To clarify the detailed mechanism maintaining the appropriate amount of Pmr, we need to clarify the mechanisms underlying this regulation.

Our results clearly indicate that TurB increases in the H-NS family protein pool in cells as TurA decreases, suggesting a functional relationship between these two proteins. Actually, our previous transcriptome analyses using gene disruptants implied that TurA and TurB play rather complementary roles as global transcriptional regulators in response to plasmid carriage, because the similarity of the TurA and TurB regulons was comparatively higher [16]. This relationship is similar to that of MvaT and MvaU in *P. aeruginosa*, where MvaU binds almost the same regions as MvaT on the genome and can partially complement the repressing function of MvaT in an *mvaT*-deficient strain [27, 28]. Considering that MvaT and MvaU affect each others amounts [27], the amount of TurA and TurB can be changed in *turB* and *turA* disruptants, respectively. On the other hand, in the pCAR1-harboring KT2440 cells, the relatively high expression of Pmr had no effect on the amounts of TurA and TurB, implying the possibility that Pmr works relatively independently. This is consistent with our previous observation that the Pmr regulon differs significantly from those of TurA and TurB [16]. The function of Pmr appears to differ from that of Sfh, which can replace the function of chromosomally encoded H-NS and acts as a molecular backup [29, 30]. It is possible that the expression level of Pmr is not affected by the absence of TurA or TurB in contrast to Sfh whose protein amounts increased when H-NS or its chromosomally encoded homolog StpA was absent [31].

In addition to Pmr, pCAR1 contains genes encoding two other highly transcribed NAPs: Phu, which is an HU subunit homolog, and Pnd, which is an NdpA homolog [32]. When *pmr* and *phu* or *pmr* and *pnd* were simultaneously disrupted, stability and transfer frequency of pCAR1 were significantly decreased, suggesting that Pmr acts synergistically with Phu and Pnd [32]. Quantification of expression levels of Phu and Pnd, as well as their functional characterizations, is necessary to clarify the whole landscape of cooperative functions of NAPs encoded on pCAR1.

## Conclusions

Our results revealed that the expression levels of TurA and His-tagged Pmr were one order of magnitude higher than that of TurB when pCAR1-free or -harboring KT2440 cells were cultured under the same conditions. Furthermore, TurA and His-tagged Pmr were highly expressed throughout the whole growth cycle with different translational profiles, while TurB was only detectable in the early stationary phase.

Information on protein expression levels provides a basis to explore the detailed manners of the functions of H-NS family proteins, i.e., how they form homo- and hetero-oligomers on DNA. Recently, preference of target DNA sequences of MvaT in *P. aeruginosa* [25] and the structure of dimerization/oligomerization domain of TurB [33] were reported. We are now performing kinetic studies on the DNA-protein and protein-protein binding properties of TurA, TurB, and Pmr. Together with the current results, these results will clarify the molecular basis of how these proteins form oligomers on the *Pseudomonas* genome.

## Additional file

Additional file 1: Representative western blotting results of the three H-NS family proteins. The amounts of TurA, TurB, and His-tagged Pmr were monitored at various points in the growth curve of KT2440 (A), KT2440(pCAR1) (B), and KT2440(pCAR1pmrHis) (C). Results of western blotting analyses using anti-TurA (left panels in A and B), anti-TurB (right panels in A and B), and anti-His antibodies (C) are shown in the black boxes. The loading amounts of whole cell protein were 80 μg for KT2440 and KT2440(pCAR1) and 40 μg for KT2440(pCAR1pmrHis) per lane. Specific amounts of purified His-tagged proteins (shown in red) were used to quantify the number of protein molecules. For each strain, 5 μg of whole cell protein were loaded and detected by anti-RNA polymerase α subunit (RNAP) antibody (green boxes) as a loading control. (PDF 83 kb)

## Abbreviations

Cm: Chloramphenicol
ECL: Enhanced chemiluminescence
His: Histidine
H-NS: Histone-like protein H1
Inc: Incompatibility
Km: Kanamycin
LB: Lysogeny broth
NAPs: Nucleoid-associated proteins
OD_600_: Optical density at 600 nm
PVDF: Polyvinylidene difluoride
qRT: Quantitative reverse transcription
