# Supplementary material for: Growth phase-dependent expression profiles of three vital H-NS family proteins encoded on the chromosome of Pseudomonas putida KT2440 and on the pCAR1 plasmid
Source: BMC Microbiol. 2017 Aug 29;17:188. doi: 10.1186/s12866-017-1091-6 (PMC5576294; doi:10.1186/s12866-017-1091-6)
Supplement: Additional file 1: — Representative western blotting results of the three H-NS family proteins. The amounts of TurA, TurB, and His-tagged Pmr were monitored at various points in the growth curve of KT2440 (A), KT2440(pCAR1) (B), and KT2440(pCAR1pmrHis) (C). Results of western blotting analyses using anti-TurA (left panels in A and B), anti-TurB (right panels in A and B), and anti-His antibodies (C) are shown in the black boxes. The loading amounts of whole cell protein were 80 μg for KT2440 and KT2440(pCAR1) and 40 μg for KT2440(pCAR1pmrHis) per lane. Specific amounts of purified His-tagged proteins (shown in red) were used to quantify the number of protein molecules. For each strain, 5 μg of whole cell protein were loaded and detected by anti-RNA polymerase α subunit (RNAP) antibody (green boxes) as a loading control. (PDF 83 kb) [file 12866_2017_1091_MOESM1_ESM.pdf]

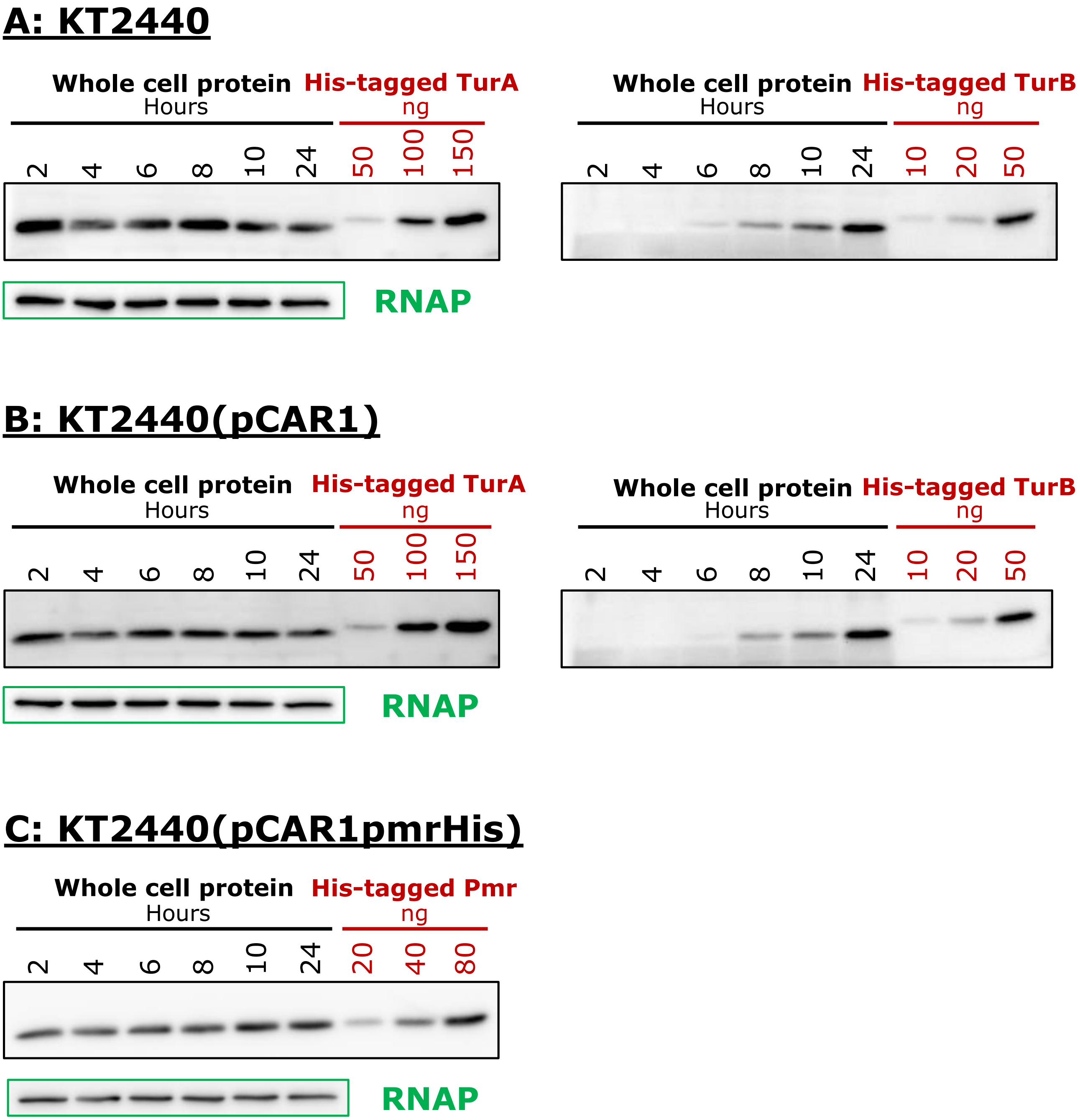

Additional file 1 Representative western blotting results of the three H-NS family proteins

The amounts of TurA, TurB, and His-tagged Pmr were monitored at various points in the growth curve of KT2440 (A), KT2440(pCAR1) (B), and KT2440(pCAR1pmrHis) (C). Results of western blotting analyses using anti-TurA (left panels in A and B), anti-TurB (right panels in A and B), and anti-His antibodies (C) are shown in the black boxes. The loading amounts of whole cell protein were 80 μg for KT2440 and KT2440(pCAR1) and 40 μg for KT2440(pCAR1pmrHis) per lane. Specific amounts of purified His-tagged proteins (shown in red) were used to quantify the number of protein molecules. For each strain, 5 μg of whole cell protein were loaded and detected by anti-RNA polymerase α subunit (RNAP) antibody (green boxes) as a loading control.
